# American Dermatological Association

**Published:** 1882-11

**Authors:** 


### Society Reports.

### ARTICLE VII.

AMERICAN DERMATOLOGICAL ASSOCIATION. Proceedings at the Sixth Annual Meeting, held at Newport, R. I., August 30 and 31, and September 1, 1882.

### FIRST DAY'S SESSION.

A business meeting was held at 9:30 o'clock, with closed doors, at the commencement of the morning session. At ten o'clock the regular session began with the Annual Address by the president, Dr. Hyde, of Chicago, which he read, as follows:

*Gentlemen of the Association:* It becomes my agreeable duty to utter a few words of greeting and welcome at the opening of the sixth annual meeting of the American Dermatological Association.

The mere announcement of this fact suggests to us that this is an occasion for congratulation. The survival, for even this relatively brief period of time, of any special Association of American physicians, ought in some measure to demonstrate that it had occupied its ground and was giving promise of a future. But when such an Association is in position to give a good account of work actually accomplished, surely this supplies both a reason for its existence and an argument for its perpetuity.

Five years do not, it must be admitted, furnish a measure of time on which to predicate maturity, but five years of dermatological work in these days of intellectual activities and scientific appetite, five years of this work done in an Association without a predecessor, and in the absence of a precedent, five years of such work in a country where the special cultivation of the field is of comparatively recent date, this may indeed be a source of profound satisfaction. During these few years of its existence, this Association has been in actual session for less than a fortnight altogether, yet the labor which it has stimulated, the influence which it has exerted, and the position which it has attained, both in this country and abroad, as a body of scientific colaborers, all these are to be estimated by another standard than that merely of time. Were its work to be concluded with this session, and the last paper to which it should listen be that read on the closing day of this meeting, I should find no fewer reasons for congratulating you on the results accomplished in these five years of labor. No man could sit down to-day to write the history of the progress of medicine in this country for a similar period, and omit to mention the contributions to its literature directly and indirectly made under the auspices of this Association. No man could sit down to-day to trace the progress of medical education for a similar period in these United States of America, and be silent upon the increased attention given to the study of cutaneous diseases in our schools of medicine. A large part of this, indeed, is due to the presentation, on the part of this Association, of the claims of such study to a higher recognition, based not merely upon the attractiveness and interest which it always awakens, but upon its essential importance.

Five years ago, many of our recent graduates in medicine recognized eczema only as a vesicular disease, and their armamentarium for cutaneous maladies was restricted to arsenic for internal use and the benzoated oxide of zinc ointment for topical employment. To-day, many even of our undergraduates who have not yet passed their final examination for a degree, can distinguish between the varieties of lupus, can point out the lesions of pediculi on the skin of the syphilitic patient, and can affirm that this or that eruption is of artificial origin. I do not think that it is claiming too much to hold that for this improvement and promise, the influences exerted by this Association are in part responsible.

Reviewing, then, even for this relatively brief period of time, the results actually accomplished by the American Dermatological

Association, it occurs to me that we can find reason for congratulation in the following facts:

First. The ground has been occupied. The Association has demonstrated its existence and its capabilities of usefulness to the medical profession of the country. It is here recognized as solely assuming to limit its membership to experts in the field which it occupies; and its proceedings have attracted an attention and been awarded a meed of authority largely proportioned to these conditions.

Second. It has avoided dangers which beset the path of many ventures and experiments. It has assumed no financial obligations which it was not in position to redeem. It was, at the time of its first organization, fortunate in the adoption of a sufficiently simple scheme of self-perpetuation and procedure, in consequence of which it has been enabled to escape the burden of an elaborate and complicated system of rules. It was, moreover, in no haste to enlarge its membership, wisely preferring that its portals should be opened to those only who had previously demonstrated their special fitness for such honor. It has also escaped the dangers incidental to bodies composed of an equally limited number of men acknowledging a community of interests and actuated by similar motives. Its discussions have never lacked virility, and that degree of divergence of opinion and practice which will always characterize the disciples of true science, so long as there subsists the recognized proportion between the known and the knowable, the attained and the attainable. That upon which we all agree, probably bears an increasing ratio to that upon which we all differ, and with this every scientific Association must perforce for the time be content. When the representatives of these bodies are upon all points agreed, then the reasons for their corporate existence are reduced to the level and scope of a mere sentiment.

Third. The transactions of the Association bear witness to the creditable amount and character of the work performed by its members, both within and without the limits of its sessions. The line of thought in which your attention is thus directed, has been in part suggested by the fact that you are this year to receive the consolidated report of the chairman of the committee on statistics, covering the entire period during which those statistics have been in course of collection. This report will be fitly presented by the first president of the Association, to whose address on the occasion of its first meeting we are indebted for the suggestion, subsequently formally adopted and set in force, relative to the organization of that committee. This report will inform you that we have collected the statistics relative to no less than sixty thousand cases of cutaneous disease, a larger number than has been heretofore tabulated in any country. The value of these figures, collected as they have been from many and distant parts of the same country, can scarcely be overestimated. It is difficult to believe that the permanent chairman of this committee, when his project first assumed definite shape, could have realized how rich would be the results obtained within the time thus far devoted to the work.

Making exception of the remarks made introductory to the last meeting, your presidents have on four occasions made annual addresses, whose value I need not determine for you, who have not only listened to them, but have also read them, as they were printed in full in the annually published volumes of transactions. Glancing over the other papers regularly presented to the Association, we find that they can be properly assigned to each of the several classes, that of the Neuroses alone excepted, which we have recognized in our nomenclature and classification of diseases of the skin.

Of those not assignable to such classes, three papers relate to the general subject of anatomy; three to general pathology; two to general etiology; three to general, and two to special therapeutics of the skin.—(13).

In Class I, including disorders of the glands, one paper has been presented upon seborrhœa, and two upon molluscum sebaceum.—(3).

In Class II, of the inflammations, we have thirteen papers to tabulate. One, on dermatitis traumatica; four, on dermatitis venenata; one, on herpes progenitalis; one, on psoriasis; one, on lichen planus; two, on eczema; one, on prurigo; one, on acne; and one, on impetigo herpetiformis.

In Class III of the hæmorrhages, we have to record a single paper.

In Class IV, of the hypertrophies, we record one paper on papilloma, which may be properly registered under the title of verrucca; three, on hirsuties; two, on scleroderma, and one, on rosacea.—(7).

In Class V, of the atrophies, we record one paper on vitiligo, and two on atrophia pilorum propria.—(3).

Class VI, of the new growths, includes the largest number of subjects to which our attention has been thus directed. The figures are: angioma, 1; angioma pigmentosum et atrophicum, 3; lymphangioma, 1; scrofuloderma, 2; syphilodermata, as to the chancre, 1; as to the nature of the constitutional disease, 1; as to its cutaneous and other lesions, 9; lepra, 4; and sarcoma, 2. —(24).

Class VII, of ulcers, includes a single paper.

Class IX, of the parasitic affections, covers five papers, all relating to the vegetable parasites. There are seven papers, including one report, on subjects not embraced in our classification. Of these, two relate to diseases of the mucous surfaces; two, to ainhum; one, to pityriasis maculata et circinata; one, to a rare form of multiple cutaneous tumors, accompanied by severe itching; and one, to a singular form of tuberculo-vesicular lesions of the skin. The total number is sixty-seven.

Many of these contributions were illustrated by the exhibition of oil paintings, sketches, pathological specimens, or sections mounted for microscopical examination. In a few instances, patients affected with diseases under discussion, have also been presented. Medicinal substances and apparatus for the relief of cutaneous disorders by topical application, may be named in the same connection.

With few exceptions, these papers have been published in dermatological or general journals of medicine, and taken together they constitute a mass of literature, which the American School of Dermatology may claim as distinctively its own, and for which the scientific world is richer. It represents the advance of knowledge in this department for the period which it covers.

Mention should also be made of a series of minor papers or reports, embodied annually in the report of the chairman of the Committee on Statistics, many of which, either wholly or in part, have appeared in our transactions.

The regular annual statement of the contributions to dermatological literature, by the members of this Association, and at the outset by others also who had in the past to some extent exhibited an interest in this or allied subjects, has demonstrated its value. Some of the titles here collected are very naturally represented also upon the programmes of our meetings, but this is true of less than one-seventh of the entire number.

Here we have tabulated more than three hundred original papers, clinical notes, reports of cases and analyses of dermatological statistics; more than one hundred reviews and critical notes of treatises on diseases of the skin; twelve atlases, lithographic and photographic, depicting cutaneous disorders; twelve volumes of original authorship, inclusive of one translation; fifty series of digests and periodical reports of the progress of dermatological science; and more than sixty lectures, clinical and didactic, on the same subjects.

The larger part of this work has been accomplished by members of this Association, under its immediate influence. All of it has been the fruit of American research and observation.

Fourth. A final cause for congratulation may be recognized in the fulfillment of the other purposes of the Association, as these are set forth in the address of its first president, read at our first gathering. These relate to the fostering of an acquaintanceship, to say nothing of the resulting friendship, between gentlemen of distant parts of the country, each of whom, however otherwise made familiar with the special work accomplished by each other, would, without the facilities afforded by the meetings of this Association, be to that extent isolated in his professional labor and interest.

With this fair showing for the past, we naturally turn to the future. It is impossible to doubt that this Association gives a promise of usefulness to its members, and in a scarcely less degree to the medical profession of the country, whose realization will be indeed precious. By pursuing the same straight and simple path which it has heretofore recognized as safest, making sure of its facts and securing the results, with a cool disbelief for that which can not be shown to be worth accepting, it surely needs no great change in either its purpose or policy to reach the desired end.

We can, however, with profit, constantly ask in what directions and by what methods may we further develop our resources and enlarge our opportunities ?

There are a few suggestions which occur to me in this connection, no one, probably, new to any of you, all of them having been, I think, at various times the subjects of discussion.

The first relates to giving some prominence upon the annual programme to a definitely fixed time, to be specially devoted to the exhibition of specimens, mounted sections, medicinal substances, drawings, etc. We have already made a step in this direction. By giving more attention to this allotment of time, it seems probable that not only the number of articles annually exhibited might be increased, but also that medical gentlemen of recognized position and attainments, even though not members of the Association, might be glad to make occasional contributions of this character. The time may not be distant when the Association will be ready to preserve the specimens thus gathered in a pathological collection. I do not know that we are yet ready to invite respectable drug manufacturers to exhibit to us the preparations placed by them upon the market, for use in the treatment of cutaneous diseases, but I am confident that we have faith in that American enterprise and ingenuity which have solved so many of the problems with which the old world had previously taxed itself in vain. Upon a single occasion, you will remember that I took the responsibility of inviting a communication of this character, which was received by the Association with a vote of thanks.

Second. It has at times occurred to me that a clinical, pathological or therapeutic portfolio might be annually opened at these meetings with advantage to us all. Such a portfolio might be permanently entrusted to the charge of a single member, or its holder might be annually selected for one year's service. It should contain voluntary or solicited contributions from the various members of the Association, of a shorter character and less elaborate form than those expected of papers regularly announced upon the programme. These would naturally be memoranda of clinical observations, brief illustrations of new or old therapeutic measures, notes on the coincidence or succession of cutaneous diseases, etc., fragments which considered either *per se*, or in mutual relation, would prove interesting and often valuable. I have been struck with this when corresponding with members of the Association, some of whom have been requested at random to communicate their experience upon certain points. Even the negation of such experience may, in such cases, prove a matter of importance. We have not rarely had members present at these meetings who have not contributed papers. This, certainly, has not been due to the fact that they had not made observations of interest and value during the year, for there is no physician in active practice who cannot make a year's study of his cutaneous experience valuable and interesting, but because there was lacking either the patience or the opportunity requisite for the preparation of a formal paper. The portfolio I have suggested would preserve these less formal contributions and prove, eventually, important as a compendium of facts for reference.

The third suggestion was originally made in writing, and addressed to the Association by one of its members. It contemplates the appointment of a committee charged with collecting information on certain definite subjects. By the measure proposed, the effort of the Association would be systematically directed in certain channels of observation. However freely we may admit the futility of attempting to solve any scientific problem by the appointment of a committee, a step in the direction desired might unquestionably be taken, and might lead to very practical results. For example, it has been a matter of discussion in this body, whether the subjects of acne were or were not habitually the victims of dyspepsia. For the past two years I have recorded opposite every note relative to cases of acne in which my advice has been sought, a memorandum containing the ascertainable facts in this connection. With the results obtained, we are not at present concerned, but they serve to illustrate the value of similar observations conducted on a much larger scale. In some such way as this an effort might be made to obtain a knowledge of the natural history of many disorders not dangerous to life, each member engaging to observe a definite number of untreated cases of any given disease for an agreed period of time.

The last consideration respects the increase of membership in this Association. None of us are willing to see a name added to our rolls which is not worthy of such a place. Each of us surely is willing to place there every name deserving of such inscription. We are called upon, it seems to me, to act in this matter with due conservatism, on the one hand, and with a wise discretion on the other, recognizing the need of that increment which the purposes of this organization must eventually require for their fulfillment. Many of the physicians of this country are annually perfecting their education in special departments of medicine by foreign study and observation, and with the enlarging clinical advantages of the metropolitan centers of our own country, are steadily acquiring a position which must claim our attention. Of these there are probably some who would not choose to apply for admission to our ranks. It may be well for us at no distant day to authorize the council, or a committee specially selected for that purpose, to propose the names of suitable candidates, who would thus be presented for our suffrages without laboring under the disadvantages of relying for support upon a single proposer, or of being themselves applicants for the privilege of admission.

In conclusion, I may be permitted to express the hope that he who shall, in five years from this time, pass under review the achievements of this Association during the first decennial of its existence, may find it his agreeable duty to chronicle a proportionately larger measure of good results accomplished, and to point to no less brilliant prospects in the future.

---

Dr. C. Heitzmann, of New York, made some remarks with reference to Studies on Myxo-Angioma of the Skin, Clinical and Microscopical. (Published in *The Medical Chronicle*, Nov., 1882, p. 90.

Tumors of the skin, termed angioma, or vascular, or erectile tumors, are of frequent occurrence. They appear either as purplish spots, sharply marked from the neighborhood, or as dark-red elevations, sometimes distinctly pedunculated. These tumors are more or less compressible; they, in the majority of the cases, are not congenital, but appear in early childhood, remaining stationary or slowly extending over the surface of the skin. Congenital angioma is known as nævus flammeus. It is sometimes pigmented, and is usually freely supplied with hairs.

We distinguish three varieties of angioma,—the simple, the lobular, and the cavernous angioma. In the first two forms, arterial, venous, or capillary blood-vessels may prevail, while in the third form, the veins are mainly involved. In simple angioma we find a more or less uniform distribution of the blood vessels, and between them, as a rule, myxomatous connective tissue, establishing the diagnosis myxo-angioma. Lobular angioma is composed of coils of blood-vessels, which are held together by a delicate fibrous connective tissue, there being between the coils coarse fibrous connective tissue. Cavernous angioma is venous in nature, and represents an imitation of the structure of the cavernous bodies of the penis. Angioma, though of a strictly benign nature, sometimes breaks open by ulceration and leads to hæmorrhage, which becomes alarming only in the cavernous variety. The latter is sometimes painful in a high degree.

As therapeutical measures, excision, cauterization, vaccination, and the artificial ulceration by irritating remedies have been resorted to. Small tumors are best destroyed with fuming nitric acid transferred on the point of a hard wooden stick, which is simply saturated with the acid. Pedunculated tumors are cut off by a pair of curved scissors, the cut surface being immediately afterwards touched with liquor ferri sesquichloridi. Larger tumors need excision with the knife. Electrolysis serves in many instances for the destruction of the blood-vessels, if the negative pole of a constant electric current is brought to bear upon the newly-formed tissue by means of numerous applications with needles. Whether the success thus obtained is permanent will be demonstrated by observation of the tumors operated upon for a number of years. The method is of too recent a date to allow any positive statements as to its value.

Myxomatous tissue, which is largely involved in the construction of myxo-angioma, appears as medullary, reticular, lymph, or adenoid tissue, and also in the form of a tissue resembling that of the thyroid body. Myxomatous tissue is present in large quantities throughout the animal body in the earliest stages of embryonic development. It also forms exclusively the tissues of transient service, such as the placenta and the umbilical cord. In the adult, myxomatous tissue is met with only in the vitreous body of the eye, in the lymph-tissue, including the layers widely spread over the so-called mucous membranes, erroneously termed adenoid tissue. It is most prevalent in the alimentary tract and in the mucosa of the uterus.

The reticular variety is the most likely to form soft, benign tumors, termed "myxoma," and this variety constitutes also the basis-substance between the blood-vessels in the myxo-angioma. Here the blood-vessels are constructed of large endothelia, and are characterized by a comparatively wide caliber. In rare cases, pigment is found in myxo-angioma, which indicates a tendency toward the formation of a malignant type, the sarcoma (myeloma), especially the melanotic myeloma. If such a change takes place, the attempts at eradication of the tumor are usually not successful.

DISCUSSION.

Dr. Piffard said that the treatment of these growths by electricity had in his experience proved unsatisfactory; he had found them very difficult to destroy, and very likely to return. Paquelin's cautery and the hot needle had been more successful in his hands, although several applications may be required. These tumors sometimes shrink and disappear of their own accord. When the growth is stationary or becoming smaller, it should not be meddled with, but where it is increasing in size some operation is required; it is in this case, also, that a re-growth often recurs.

Dr. White enquired if the lecturer could explain the tendency to ulceration which sometimes is manifested; whether inflammatory or due to stasis, and why it occurs least in advanced life?

Dr. Heitzmann believed that it was inflammatory originally, leading to engorgement, strangulation and stasis, although there are no positive observations upon this point. The only explanation of the latter fact is that late in life, the structure of the growth changes; the intervening tissue becomes more fibrous and less myxomatous.

Dr. White stated, with regard to the removal of nævus, especially the form known as spider-cancer, that the destruction of the central knob with electrolysis, is often sufficient to produce permanent relief; but in other forms it had failed. He had repeatedly tried to destroy them by this means, but had never succeeded.

Dr. Taylor spoke of ulceration and hæmorrhage, and mentioned two cases in which nothing would stop the bleeding. He inquired if Dr. White had seen such cases get well?

Dr. White said that he had.

Dr. Taylor asked if they were large?

Dr. White said, no, not very large.

Dr. Taylor stated that he had now such a case under his care in which the growth covered the entire half of a child's head; the face especially was involved.

Dr. Piffard said that in his previous remarks, he had only intended to refer to the large, deep-seated growths about the face and lips as being so hard to cure; the superficial varieties he had not found difficult to treat.

Dr. Taylor said that in one of the earlier meetings of the Association he had reported cases of angioma pigmentosum et atrophicum which were still under his care at present. The first case had progressed since that time; of the tumors over the face, the growth at the angle of the eye has gone on increasing and now had become very vascular and completely covered the eye. The growth is pediculated and looks like lobules of tissue. The general object of treatment had been to reduce the size of these masses; several surgeons had attempted to remove it but without success. Liquor potassæ (ʒij to ℥j) was now being tried, and apparently with good effect; the tumor is getting decidedly smaller. In the second case, the tumor still exists upon the cheek.

He insisted upon the peculiarity in the appearance of the spots in these cases. At first there appears a litte pin-point elevation which increases and becomes more or less linear, and decidedly red in color, and afterwards leaves a pigmented spot; this repeats itself, the lesions appearing in crops. He could not say positively that the pigment did not come first; in some cases the growths wither away, in others they develop into angioma.

Dr. Duhring's case should not be mentioned in this connection. It was an old case, and the pigment was distinctly a sequela and not of original formation. As Dr. Fox has had a number of cases, which will probably be reported at the next meeting of the society, the speaker would defer further remarks upon the subject until then.

Dr. Piffard observed with regard to pigmented deposits succeeding vascular congestion, that in almost all cases where maculation occurs as a pathological condition, it is the result of hæmatine deposition, and it is probably only in a few exceptional cases (as in the eye) that we have genuine ooze deposit of melanine.

Dr. Taylor suggested that possibly the lecturer could explain why we have in some cases a local increase of circulation, producing a red spot, which afterwards becomes pale and yellow,—and no epithelium near it.

Dr. Heitzmann asked if Dr. Piffard could give any method of distinguishing between hæmatine and melanine?

Dr. Piffard said, yes. Hæmatine is easily recognized by tests that are well known; it is exceedingly soluble; melanine is not soluble, etc. Robin pointed out the distinction ten years ago, in the *Journal de l' Anatomie et de la Physiologie.*

Dr. Heitzmann inquired if melanine discoloration occurs after a hæmorrhage.

Dr. Piffard said that he did not understand how melanine discoloration could occur after extravasation of blood. Even the old brown spots on the legs after prolonged ulceration, are really due to hæmatine, but the hyperpigmented discolorations of vitiligo are probably due to melanine, and not to the blood discoloration, the pigment being heaped up more in some cells and less in others.

Dr. Heitzmann merely inquired if there exists any means of distinguishing them in practice.

Dr. Piffard replied that the means of distinction are very clearly laid down in the article by Robin, to which he had referred.

Dr. Heitzmann said that it is a very important question for the dermatologist. The test is in the color of the negro, which is due to normal pigment in the cells of the skin. In explanation of the excess of pigment in the dark races, it may be said that it is the result of heat, but it is seen that men who work in the heat constantly, as in foundries, the heat does not produce such an effect.

Dr. Atkinson believed that heat will produce pigmentation, chloasma, for instance. He had seen the statement that large patches of chloasma occurred on the buttocks in women who sit over charcoal furnaces in France, and he had seen similar cases.

Dr. Hyde said that he had been struck with the remark made by Dr. White, that freckles and "tanning" occur in places not exposed to the sun, but simply from the influence of the water. In the bath houses at Chicago, the women complain of the effect upon their complexions, although the bath houses are shut in and covered.

Dr. White remarked that he had already called attention to the difference between the effects of the sun on the land and water; and in different persons; some will burn without any pigmentation whatever, others will tan without burning. The difference does not depend upon the amount of burning but to personal peculiarity. In some it would seem as if the pigment cells were formed under the direct influence of the sun, without inflammation.

Dr. Piffard claimed that this confirmed what he had said a few moments ago, that the coloring matter of the skin is melanine, which may be increased or altered by the effects of the sun, by causing direct hypersecretion of these cells, without altering the circulation.

Dr. Hardaway had noticed a similar discoloration of the skin in old people who sit around the fire.

Dr. Rohé said that he had a few patches of leucoderma in his hands. A few years ago, while at sea, he was exposed to the sun and became much tanned in consequence, but these patches upon his hands became red and painful, but did not tan. Afterwards the burning passed away, and the spots became pale again, in marked contrast to the surrounding skin.

Dr. White further remarked that pigmentation may occur as the result of hyperæmia, and also without preceding hyperæmia. For instance, in morphœa, pigmentation may be said to follow congestion, but in vitiligo there is never any inflammation or increased flow of blood to the parts. He could not see how Dr. Heitzmann would be able to support his view, in this case, that pigmentation is in any way dependent upon the coloring matter of the blood.

Dr. Heitzmann said that if the particles of living matter are saturated with the coloring matter of the blood, pigmentation will be effected without local increase in circulation. It is noticeable that chronic inflammation in the two hollow organs, the stomach and the bladder, often leads to a deposit of pigment in their walls, which shows that a long continued increase of blood in the tissues will produce pigmentation. The remarks of Dr. White show that there are two kinds of pigmentation.

Dr. Taylor referred to the case of exposure of the face to the rays of the sun when the skin becomes brown, but all above the line where the forehead is protected by the hat it remains pale. He did not believe that there can be such a line of demarcation of hyperæmia.

Dr. Heitzmann.—This carries out the observation of Dr. White; the sun must have something to do with it.

Dr. Piffard said there must certainly be two kinds of pigmentation, that due to hæmatine and that due to melanine.

Dr. Hardaway observed, in conclusion, with regard to the electrical treatment of angioma, that, in "spider cancer" his experience agreed with that of Dr. White. He had treated it successfully by electrolysis. In one case, a tumor on the lip, the size of a half-dime, disappeared and did not return. On the other hand, some of these cases must tend to spontaneous cure, for the most simple remedies have been used, and were followed by a recovery; for instance, the oil of ergot has been used, and a cure followed, though probably not as an effect.

The absence of scar after electrolysis, and the perfect control which is had over its action, renders it much more useful than acid in the treatment of angioma. With regard to acne rosacea, however, there is a tendency to return if the cause of the disease be kept up; in a number of cases in which he had also been able to treat the cause, he had succeeded in curing the disease, and it had not returned ; nevertheless, the use of electrolysis will diminish the tendency to the formation of blood-vessels. He prefers to use a large number of needles, as by this means he was able to obtain better results; he also observed that the electrolysis lessens the rosacea better when combined with other treatment, at least this had been his own experience.

Dr. James Nevins Hyde read a paper entitled *

A CLINICAL STUDY OF DERMATITIS PAPILLARIS CAPITIS, the first vice president, Dr. William A. Hardaway, of St. Louis, having been called to the chair.

DISCUSSION.

Dr. Taylor said that he had a case of this kind under treatment at present. The disease first came under his observation eight years ago, in a case at the Presbyterian Hospital in New York. It was then pronounced keloid, no history having been obtained. It was operated upon by Dr. Brinton, who removed the entire mass. Upon examining it microscopically it was found not to be keloid, but an inflammatory mass. Photographs of the sections were made, and are still in his possession.

Dr. White inquired what was the maximum time required for the development of the disease.

Dr. Hyde replied that it took a year and a half in one case; from his own observations, he would consider the briefest period to be at least six months.

Dr. Taylor said that he had a case now under observation, in which six months had passed, and it had apparently not reached its maximum yet.

Dr. Hyde, in reply to a question, remarked that no lesions appeared on the face or on any part of the scalp except the portion referred to; elsewhere there was complete immunity.

Dr. Piffard said that one of the most interesting cases he had seen was one presented by Dr. George Fox at the Pathological Society, it presented the character described by the lecturer, and the question with regard to the diagnosis had been, was it syphilis? was it keloid? or was it kerion?

---

* Published in the *Journal of Cutaneous and Venereal Diseases*, Vol. I, No. 2, p. 33.

Dr. Duhring recalled a case that he had seen a few months ago; he had only seen it twice; it corresponded with the description given by Dr. Hyde. The patient was a mechanic, thirty years of age; the disease had existed in the position indicated for a year and a half. It gave him very little inconvenience; it was unaccompanied by itching or pain; the only reason for seeking advice was the slight trouble in combing his hair. When he came under observation the patch was the size of the palm, say 4x 4½ inches. It was composed of pin-head sized to pea-sized papulo-pustular lesions, disseminated and confluent. The disease was recognized as a rare form, and although it gave the impression of some induration like keloid, the diagnosis of keloid was not made. He at once recognized it as a case of this disease, which was the first he had seen. The clinical features were so marked that the case made quite an impression upon him; he concluded that it was an inflammatory affection, of an extremely slow course. The hairs upon the affected part were in some instances wanting, in others they were still in the follicles, but they were atrophied and easily plucked out; they were not twisted nor broken; here and there were a few divided, but there were not many so. Alopecia existed to a certain extent, but it looked as if complete alopecia would follow if the disease were to continue. Keloid was excluded from the diagnosis by the clinical features; kerion by the microscopic examination, by which no parasite could be detected.

Dr. Atkinson said, it seems that notwithstanding how obscure this disease may be, there is one point in the pathology that we can be very certain about; it is a folliculitis. This is shown by the appearance of the inflammation; it is shown by the peculiar discharge. If we have an inflammation of the skin, a catarrhal inflammation, we have the gummy, sticky, transparent discharge; and if the catarrhal affection involves the hair follicles very superficially, we also have this peculiar discharge in kerion, when we have this form of folliculitis. Liquor potassæ will also excite the same form of inflammation. A German authority describes this form of folliculitis and papillary outgrowth, as the result of administration of iodide of potassium. (*Vierteljahreschrift.*) He is sure that it is an inflammatory trouble, but whatever there is of it, is due to inflammation of the follicles, unless we may imagine it to be due to long-continued hyperæmia, and to result from hypernutrition; the coloration of these spots is of the same character. There is slight exudation, such as is found in old chronic eczema.

Dr. Heitzmann inquired the nature of the fluid which could be squeezed out.

Dr. Hyde said that at first it is clear lymph, and afterwards bloody.

Dr. Heitzmann corroborated what Dr. Atkinson had said. He referred to a case which had been under his care, a negro, who had the disease in the back of his neck. Upon examining it, his first impression was that it consisted in inflammation around the sebaceous glands and hair follicles. It occurred to him to ask the man if he had been in the habit of rubbing his scalp at night, and on his next visit the patient said that it was so, that he had been rubbing his head at night against a rough neck-band of his shirt. The second case, a Jewish merchant, exhibited the same lesions as those referred to in Dr. Atkinson's remarks. Here it was also found to have been caused by rubbing the back of the neck against a rough collar. This case he did not see again.

Dr. Taylor said that in his cases, at first, he used a slightly stimulating treatment; later, he applied liquor potassæ. He hoped that his cases would escape without a scarred appearance; he did not care if this treatment does destroy the hair follicles, as they will be destroyed anyway.

Dr. Hardaway said that in a case he had examined, he had discovered the primary lesion to be tuberculo-pustular, and had observed this peculiar secretion. He had had two cases besides, where the peculiar appearances so well described, were all present, except that it had not reached the keloid stage. The disease was in the region rubbed by the shirt collar. As this disorder is not found elsewhere, and as it would not occur here except for good cause, he was inclined to regard, as the efficient cause, the rubbing and irritation of the collar.

Dr. Hyde replied that this explanation seems like a very simple and easy way out of the difficulty, but it did not quite satisfy him. He had never seen a simple case of eczema which might be mistaken by experienced observers for epithelioma, kerion, or keloid, nor had he seen such keloid growths from chronic eczema. Moreover, the disease also occurs further up on the scalp, where the collar cannot reach. He thought while Dr. Heitzmann was speaking that he was about to say that it was due to scratching at night, with the fingers, while partly asleep. This might be the cause of the irritation, but the pathology of the disease is not well understood.

Dr. Hardaway said that in the negro the disease was lobulated.

Dr. Duhring said that in his cases there had been no such irritation from the collar to cause the disease.

Dr. Hyde, in reply to a question, said that he thought the disorder was sufficiently well characterized, to warrant calling it a distinct disease.

Dr. Hardaway inquired whether the lobules corresponded with the location of the hair follicles. His own opinion was that they do.

Dr. Hyde said that it was probable.

Dr. Taylor said that in the case he had referred to, the folliculitis was marked, and a large amount of fibrinal tissue had been developed.

Dr. Hyde regretted that he had not made a microscopic examination of the secretion.

Dr. Heitzmann said that there had been no secretion in his cases.

---

### EVENING SESSION—AUGUST 31ST.

Dr. Robert W. Taylor of New York, read a paper entitled Notes on Psoriasis.*

### DISCUSSION.

Dr. W. A. Hardaway said that he thought that all were aware of the theory of Wilson, but, as Dr. Taylor had said, it is a theory which had not been accepted; and, moreover, it seemed to him, a theory totally untenable. He inquired if he correctly

* Published in *Journ. of Cut and Ven. Dis.*, Vol. I, No. 1, p. 3.

understood the lecturer to state that syphilis is the only cause of psoriasis?

Dr. Taylor said that he did not acknowledge any cause; he merely reported certain facts, and would let the members draw their own conclusions.

Dr. Hardaway remarked that it was hard to discuss the subject from this standpoint. He recalled a case of psoriasis in which the disease had appeared at the age of 65 years; but we know that hereditary syphilis does not appear so late in life. The age at which it developed is against the hereditary character of this case; he had seen it stated that psoriasis rarely appears before puberty, he had seen it himself at all ages, and often in young children, in whom he had satisfied himself that it is hereditary. He recalled an interesting series of cases. In two families of a brother and a sister with psoriasis, each with children, he could not recall the exact number of persons, but nearly the entire number had this disease. This gentleman said that his father was free from it, but that his grandfather had psoriasis.

With reference to the treatment, he would simply say that he had recently given chrysophanic acid internally in several cases, and with good results.

Dr. Duhring said that he would simply like to state that from his own standpoint he could not see that syphilis is ever the cause of psoriasis; he had simply no facts to offer in corroboration of this view. On the contrary, we all know that the papulo-squamous syphiloderm takes on the appearances so much like psoriasis that the most practiced observer finds himself hesitating about the diagnosis; but the two should be distinguished, notwithstanding. Psoriasis with its clear history so well known, has not the peculiarities of the squamous syphiloderm. From listening to the paper he was at a loss to know whether Dr. Taylor takes especial pains to distinguish these diseases or not.

There are a great many causes which may give rise to what we call psoriasis; if we look upon syphilitic manifestations as psoriasis, then syphilis may cause psoriasis; but he thought this view a very bad one to take, it confounds symptoms with disease. It would seem at least as if it would require a good deal of thought and a more exhaustive paper in its support than that theory just presented, before it can be adopted.

Dr. White thought that it would have been better if Dr. Taylor had simply stated that syphilis is one of the antecedents of psoriasis. It seemed to him that there could not be two diseases more dissimilar. The course of the disease, the fact that primary syphilis may occur in a person suffering with psoriasis, and the general results of treatment are all against this view, but if he means simply a coincidence, he merely asks whether psoriasis after syphilis may occur any more often than eczema, for instance.

Dr. Taylor said that he had not submitted any theories but simply facts; he would like others, if interested, to pursue them further. He thinks eczema an entirely different question from psoriasis. Eczema may be due to traumatism, but he had never seen psoriasis so caused. He merely observed that we are all at sea with regard to the etiology, and he had simply suggested a possible explanation.

Dr. White asked the character of the patients investigated, with regard to social position.

Dr. Taylor said that they were of all classes from the highest to the lowest. He would simply state that these cases had occurred in his experience; he merely wished to lay this fact before this body in order that further light may be thrown upon it; authorities state only negations. Dr. Duhring simply says that syphilis has nothing to do with it.

Dr. White said this is simply the point that he wanted to bring out; he thought that in twenty-five cases of psoriasis, taking them as they come, he would find a larger number of cases of syphilis than in eczema, on account of the social condition in life among which such cases usually occur; in other words, in another disease, like eczema, we would not find such a large proportion of cases of syphilis.

Dr. Taylor said that he simply gave the facts, that in a certain proportion of cases he had found this history, and he would like others to inquire into the cause.

Dr. White inquired if he could always satisfy himself with regard to the history of syphilis. If a man 65 years of age has psoriasis, what does he know about his grandfather? Did the lecturer take his cases in sequence or select those that gave a clear history?

Dr. Taylor said that he did not pick his cases. He had no desire to pick his cases to present any theory. He did not read the whole series of his cases as he did not wish to weary the society with a long report, and therefore merely gave the concrete facts.

Dr. C. Gilman Smith said that there seems to be a greater tendency from year to year to give syphilis a larger share in the causation of chronic skin diseases. He did not understand Dr. Taylor to bring this before us as a matter of conclusion but as suggestive. He would himself think that the fact that arsenic had such an effect, would lead to the view that psoriasis was not syphilis.

Dr. Taylor said that some syphilitic lesions are best treated by arsenic, as we know. He would state just here that in the young cases Donovan's solution will have more effect than in the older ones.

Dr. Robinson believed that he had asked all his cases of psoriasis this question about syphilis; and there were a considerable number of them. He was satisfied that there is not a greater proportion of cases with syphilis than he would expect to find, so that he would not conclude that syphilis is the cause. Psoriasis occurs at all ages; there are many points of difference between it and a papulo-squamous syphiloderm; there is not the proliferation of cells; and then again, if it is syphilis, it is strange that it does not appear on the palms of the hands more frequently than it does. It is common in syphilis, but he had never had a case of psoriasis which showed it. He had found cases to get well more quickly on arsenic and iodide of potassium, than on arsenic alone. He had given arsenic to a child for a month with little effect, but on adding five grains of iodide to each dose, it got well inside of another month. With reference to traumatic causes, he certainly had seen cases in which psoriasis had been excited by external irritation of the skin; in some cases it has followed vaccination, and on the spot where the child was vaccinated; it was therefore not due to some change in the whole system, but to a local change in the skin. Upon a careful consideration of the subject, he could not find any connection, or even anything upon which to base a view of there being any connection, between psoriasis and syphilis.

Dr. Rohé said that as the causation of psoriasis had come up for discussion, he would shortly report a few cases in which it followed vaccination. He had never seen any report of such a connection, and he had therefore prepared the notes for reading before this society. With regard to syphilis, he had never been able to get any evidence that any individual who had syphilis had anything to do with the generation of psoriatic children.

Dr. Heitzmann said that he thought it hardly necessary to repeat the fact that syphilis and psoriasis are entirely distinct. So long as it is merely stated as a coincidence, there is nothing to argue about. Why should not a syphilitic father have a psoriatic child?

Dr. Piffard said that there are only three distinct causes assigned for psoriasis; these are known as the suboxidation theory, the one which he favored; the parasitic view advanced by Lange, and the syphilitic theory referred to by Dr. Taylor. Now, if we compare the course of psoriasis with the ordinary course of syphilis, there is no similarity; they differ in almost every point. There is, in fact, no point in which they come in contact. Psoriasis, moreover, is a disease which was known and accurately described in very ancient times; syphilis was not, it certainly was not accurately described. If we turn to the sacred writings, Christ is stated to have healed ten cases of psoriasis (the English translation says leprosy, which is a mistranslation. Lepra is a Greek term, simply meaning scaly). Now, if syphilis did not have an ancient origin, then certainly psoriasis could not have proceeded from it. The coincidence of psoriasis and syphilis has been studied by Dr. Hyde in a paper which, it is hoped, will soon be read. The speaker had seen several cases in which primary syphilis occurred in persons with psoriasis. If psoriasis is syphilis, we would hardly expect the two lesions progressing together, the papular, and, later, the tubercular syphilide, occurring with the psoriatic lesions, which recur from day to day without change.

Dr. Taylor brings forward the statement that arsenic and mercury are useful in the treatment of psoriasis, and Dr. Robinson has found iodide of potassium useful. Of this there is no doubt, whatever. He had himself seen a number of distinct cases of psoriasis disappear under these remedies ; not cases of squamous syphilide, by no means. He had also seen, in the practice of another gentleman, the lesions of psoriasis made to disappear very promptly by the use of large doses of iodide of potassium internally, and mercury by inunction, as the sulpho-cyanide.

He thought that the whole matter might be summed up by saying that mercury and iodide of potassium are useful in psoriasis ; and, secondly, that in a certain number of cases of psoriasis there is an antecedent history of syphilis, but not more so than other skin diseases, like acne, for instance.

From Dr. White's remarks, he inferred that he thought that syphilis is very common in the lower classes ; the speaker's opinion was that the contrary is the case ; syphilis is more likely to occur among capitalists than among laborers.

Dr. Hyde said that he greatly regretted that he had not been able to find an opportunity to complete the notes which he had taken, upon which he proposed to base a paper announced in the programme in connection with this discussion, because it was based upon facts he had observed, and which were of special interest to him. He was unable to present the paper, but he could state this much: He had had under observation two cases of inveterate psoriasis, both of which were typical cases, and both of which were unmistakably infected with syphilis. One of these he saw only a few times ; the other, quite the reverse ; for six years he had this patient under observation with every recurrence, and there were many of them. He was a very obedient patient, and was willing to carry out all the instructions and experiments made upon the disease. He (Dr. Hyde) was not only familiar with the disease, but the peculiar characters and appearances of it in this patient, who subsequently contracted syphilis. Not only did he contract syphilis, but at the proper time a peculiar efflorescence appeared upon the skin, the course of which was watched with much interest, in the expectation of finding a mixed form of eruption. When it appeared it was abundant, covering him all over, but it was an unmistakably psoriatic eruption. The "trick" of the skin for six years had controlled the eruption. No difference could be distinguished between it and former eruptions; the palms of the hands and soles of the feet were spared. At the same time he was affected with adenopathy, mucous patches, and other signs of syphilis, with which we are familiar, and when the psoriasis declined an unmistakable syphilitic eruption appeared.

Dr. Taylor inquired of Dr. Robinson whether he had gone into the history of his cases with the view of ascertaining a syphilitic etiology.

Dr. Robinson said that he did find the coincidence occasionally, but not any oftener than in other diseases, and not any more often than he would have expected in a similar series of cases, say of acne.

Dr. Taylor asked particularly if he looked for it, and what was the number or .percentage.

Dr. Robinson thought that Dr. White had answered this fully. Unless we get a larger number of such cases of antecedent syphilis than we have in any other skin disease, all the argument is of no avail.

Dr. Taylor said he wished to state that he utterly repudiates the suboxidation theory.

Dr. Hardaway, referring to the exceedingly interesting cases of Dr. Hyde, inquired what was the period between the initial lesion of syphilis and the appearance of the psoriasis.

Dr. Hyde said that sixty-five days, he believed it was, from the first appearance of the sore, and his recollection was that it was fully four weeks from the time when the other eruption came out to the time when the papulo-squamus syphilitic eruption was noticed.

Dr. Taylor had noticed one fact in the treatment of psoriasis: If children are treated early and systematically, it will keep off relapses, and at least twenty-five per cent. will be cured. He thought that those inveterate cases occur because they had not been properly treated; they are difficult to cure because the skin has taken on bad habits.

Dr. Piffard inquired how many cases of children he had treated in this way, and what was the percentage of cures.

Dr. Taylor said that he did not know at the moment, but four cases he could recall which were cured by this method, and which had been kept under observation; these have not had any psoriasis for five or six years.

Dr. Piffard.—Then you have treated twenty cases, and sixteen failed?

Dr. Taylor said that he could not tell how the cases turned out, as many of them are lost sight of, but the prognosis is usually bad, as pointed out in the books. He thinks, however, that if mothers are told there is a chance, by following out the treatment, that their child may escape further manifestations of the disease, it would be better.

Dr. Hardaway recalled a case, sixteen years of age, treated by chrysophanic acid, but without any arsenic or mercury whatever. Therefore, if the case will recover from local treatment exclusively by chrysophanic acid and green soap, no conclusions can be drawn with regard to the constitutional character of the disease, and by giving internal remedies we merely take a circuitous method of accomplishing what we can do directly by local treatment.

Dr. George H. Rohé of Baltimore, read a paper on Two Cases of Acute General Psoriasis Following Vaccination.*

DISCUSSION.

Dr. Heitzmann regarded the paper as very conclusive and logical, especially the last statement, that in the psoriatic subject any irritation may produce psoriasis. He does not claim any special connection between vaccination and psoriasis. It does not need any discussion.

Dr. Hardaway had not seen any such instances of psoriasis following vaccination although he had considerable opportunity for observation. There are a number of eruptions that are apt to occur after vaccination, herpes, multiform erythema and eczema especially; the latter may be a late lesion. Vaccinal eruptions may appear at three stages: the first is during the irritation of the

* Published in *Jour. of Cut. and Ven. Dis*, Vol. I, No. 1, p. 11.

lancet, which may give rise to acute inflammatory trouble; the second stage is during the time when the vaccination poison is acting in the blood, and there may be eruptions just as from medicinal substances; then, in the third stage, during the pus formation, there may be eruptions due to something like septic poisoning. He had treated in St. Louis similar cases of eruption due to purulent otitis media; they are produced by some septic influence at work in the case.

Dr. White asked if the first case had been followed by prolonged pigmentation of the skin, also as to the size of the lesions?

Dr. Rohé replied that it probably lasted three months before it entirely faded; the primary eruption did not last more than six weeks. The lesions on the chest were not over the size of a silver dollar; there were larger aggregations of the primary eruption, but these are just the single ones.

Dr. White asked if this kind of pigmentation is common in psoriasis?

Dr. Rohé said that this dark slaty pigmentation always remained in the cases he had treated, and it remained in this case for two or three months, before the skin entirely resumed its normal appearance.

Dr. White said that this amount of discoloration seemed more suggestive of lichen ruber than psoriasis; it is a rare condition of the skin. With regard to the action of arsenic he inquired how long it had been given?

Dr. Rohé said that it was given only for a week. The reason it was stopped was because it made the patient worse. He was an intelligent person, himself a physician, and he said that he must have something else, as it disagreed with him; he could not sleep at night; he then suggested other treatment.

Dr. Taylor thought that Dr. Hardaway was correct, in a measure, with regard to the lighting up of psoriasis; he had a case occurring after an attack of scarlet fever, and in another instance after measles. He had seen a child that had fallen out of a canal boat have a slight fever, with an eruption of psoriasis following. He thought that the mere congestion of the skin might be sufficient to start the disease, or any ephemeral disturbance of of the circulation.

Dr. Piffard remarked with regard to arsenic, he had seen psoriasis almost entirely cleaned up, the redness disappearing, and cessation of scaling, before the patient had taken two doses. She took a teaspoonful of Fowler's solution by mistake. She was much bloated and it nearly caused her death, but it cured the psoriasis. He believed that we could get much better effects in some cases if the patient could stand large doses of arsenic.

Dr. White said that he had repeatedly seen cases of psoriasis show great change in a week from the effects of arsenic.

Dr. Piffard recalled some of the experiments made by Ringer and Munell upon frogs, in which large doses of arsenic were followed by shedding of the entire epithelium from the body. They are reported in the *Journal of Physiology.*

Dr. Hyde said at the conclusion of the small pox epidemic in Chicago, during last season, he had been visited in his office by the health officer of the city of Chicago, with a young lady who had what appeared to him to be a typical psoriatic eruption covering the arms to the elbows and the upper portion of her body. She gave the history of having had no disease prior to vaccination, and the appearance of this disease since then. The health officer said that out of fifty to sixty thousand cases of vacination he had only seen this one case. The speaker was surprised that there had not been more. If we take the statistics of psoriasis and compare them with the enormous number of people who were vaccinated, it is remarkable that we have not had more cases falling under our notice than there have been. He recalled a case that did not die from the effect of chloroform. A patient, was to have some teeth extracted by a dentist, under chloroform. She did not feel well and came to tell the dentist that she could not keep the appointment. Upon going out of the door, she fell dead upon the steps. Had she taken the chloroform she would have died in the chair and it would have been set down as a death from chloroform.

Dr. Rohe thought that there must be more cases of psoriasis occurring after vaccination but they have never been reported. With regard to Dr. Taylor's remark about the occurrence of psoriasis after vaccination resembling that after scarlet fever or measles, and due to general hyperæmia of the skin, he would say that in his first case there had not been any such general hyperæmia because the vaccination had not been successful.

Dr. H. G. Piffard read a communcication entitled

CALX SULPHENATA.

Dr. William A. Hardaway said that when Ringer first published the observations referred to, he had sent to London for some of the sulphide of calcium, and had been using it ever since in fair doses. While inclined to believe that it possesses this anaplastic character, there is no drug about which his opinion had vibrated so much as this. It had seemed in some cases of pustular eczema to have done good, but he had never seen any marked effect from it in recurrent furumculosis; then, again, there were cases of acne, in which his results were *nil*. This may be due to the fact that the preparations have varied in quality, but it was a fact that he had been unable to come to any conclusion with regard to its therapeutic properties.

With reference to diabetes, he recalled the fact that a paper on this subject had appeared some five or six years ago in the Detroit *Clinic*. Regarding transient swelling of the lips, he also recalled a paper published recently by Dr. Curtis, in the *Boston Medical and Surgical Journal;* he did not think that the case reported would permit the drawing of any conclusion with regard to this condition.

The hypo-sulphite of sodium had been used by the speaker very largely in small-pox; his observations had extended over several hundred cases. It had occurred to him that under the use of large doses, he had succeeded in lessening the amount of the secondary fever and suppuration, and therefore lessened materially the amount of pitting. At one period he had made this a routine treatment; it always seemed to do good; there was less fever, less suppuration, and less pitting in small-pox with the hypo-sulphite of sodium, than by the ordinary treatment.

Dr. White was just about calling Dr. Piffard's attention to the cases of peculiar swelling of the lip described by Dr. Curtis; he agreed with the last speaker that no conclusions could be drawn with regard to the use of calcium sulphide in this disease from the case reported. With regard to its effects upon glycosuria, he inquired if there had been any change in the diet.

Dr. Piffard said that he presumed that there had been no particular change in the diet, as the patient was taking the agent merely for the skin eruption.

Dr. White inquired the poisonous dose, and how does it act?

Dr. Piffard said that large doses would be thrown off, but smaller doses would be retained, and produce their anaplastic effect just like mercury. He had seen half a grain cause vomiting, and had known five grains to be given without vomiting.

Dr. White said that he had never used large doses except in furunculosis and some forms of acne. He had been almost uniformly disappointed with its effects in doses of one-eighth to one-quarter grain. He had never seen it do any good whatever in continuous furuncular disease; in acne he had also used it in all grades, but had rarely seen it produce any good effect.

Dr. Taylor asked how many cases of non-parasitic sycosis the lecturer had treated with calcium sulphide.

Dr. Piffard had not the notes with him, but believed there were from twelve to fifteen in all.

Dr. Taylor.—How long did it take to cure them?

Dr. Piffard.—About a week or two weeks.

Dr. Taylor.—Were there any bad cases, and how much time did they require?

Dr. Piffard.—Yes; they took a little longer, from two to four weeks.

Dr. Taylor.—Were any external remedies used?

Dr. Piffard.—No; he took out the hair from the follicles that were diseased, but not necessarily nor repeatedly.

Dr. Taylor.—Was there no hot water used?

Dr. Piffard.—Not anything, locally.

Dr. Taylor.—With regard to the trouble of the lip, was there any disease of the teeth?

Dr. Piffard had not asked.

Dr. Taylor said that where there are bad teeth these swellings of the lips are not very rare. He further inquired what doses were required to cure these cases.

Dr. Piffard.—One-eighth of a grain.

Dr. Taylor said that it was a remarkable result from such a cause. He agreed with Dr. White in his statements; his experience with this drug had been about the same; but he still had hopes.

Dr. Duhring said that his only experience had been with furunculosis, and he had found that the calcium sulphide acts very variably in this disease. He has had both good results and poor ones; sometimes he has been much disappointed with it. He had himself suffered from furunculosis, which refused to yield to ordinary remedies, such as arsenic, malt liquors, etc.; finally it was relieved by hypo-sulphite of sodium. This was some years ago. Now, within the last six months, he had a return of the disease, and had taken sulphide of calcium perseveringly, but had obtained no beneficial result; and it was not until he went away from home, and left his duties, that he began to improve. At the same time, he had had very gratifying results, and although he had also had disappointments, the good results were much more numerous than the failures. In acne, particularly, he had been disappointed, and had failed to get the good results which have been handed down to us during the last ten years by Ringer and others.

Dr. Smith, of Chicago, inquired as to the theory of the use of the drug in diabetes. If it has a decided anti-inflammatory power, it might be serviceable in some cases of nephritis.

Dr. Piffard said that this remains to be shown.

Dr. Robinson said that he has had great experience in the use of sulphide of calcium in furunculosis in children, and he generally had good results; where they did not recover they were generally found to be the subjects of acid dyspepsia, but certainly three-fourths of the cases did well. In acne he had also used it, but failed to recall a single case benefited by it.

Dr. Atkinson had had a large experience in suppurative disorders with sulphid of calcium; he had used it in furuncles, acne, carbuncle, and buboes, but had never been able to persuade himself that there was any good effect. In a man with general pustular eczema, the case got entirely well under the use of sulphide of calcium, but this is the only case where the results were at all good; in all the others the results were absolutely nothing.

With reference to acne, he had been much surprised by the very favorable results reported by others. He mentioned the experience of a friend of his, who was perfectly worthy of credence, who uses pyrophosphate of sodium for the same troubles that the sulphide of calcium had been recommended for, and with excellent results.

Dr. Heitzmann said that he simply agreed with the general opinion expressed by the gentlemen present that it is of no value whatever; not only had the remedy been used by him, but also by friends of his, without any result. Of course, if we pull out the hairs in cases of sycosis, we know that they may rapidly get well; he failed to see what the calcium sulphide had to do with it. If we look for the cure of furunculosis, *Fœces Canina* is an invaluable remedy, according to the experience of the older writers; or if inflammatory, it may be cured by stibium sulphuratum. Rademacher said we must have sulphur and mercury for furunculosis! We should be careful about drawing conclusions with regard to the effects of remedies. In a case of bubo that was going on to suppuration, he had put on a blister, and the inflammation disappeared, but not from the sulphide of calcium. In conclusion, he said that he did not understand that inflammation is lowered vitality; he was of the opinion that the vital changes are increased in activity.

Dr. Hyde said that he had used the preparation with the most variable results; but, he must say, with Dr. Hardaway, that he considered the hypo-sulphite of sodium solution to have a very high degree of value. He had seen the results of which Dr. Hardaway spoke, in small-pox; when given night and day, he had seen effects that were remarkable; he had observed a more rapid subsidence of the disease after its use. He must state that the sulphide of calcium as found in the drug shops is very variable in its effects.

Dr, White objected to confounding the effects of sulphide of calcium and hypo-sulphite of sodium together, as they are entirely unlike. It is probable that the better results obtained by Dr. Piffard, were due to using a better quality of the sulphide of calcium than others had employed.

Dr. Hyde, in reply to Dr. Hardaway, stated that he had used in his small pox cases from fifteen to twenty grain doses of the hypo-sulphite of sodium.

Dr. Piffard said that there were one or two original points in the paper to which he wished to direct attention. It had been observed by others that acne and furunculosis were benefited by sulphide of calcium; and he had shown that the varying effects were to be explained by the different qualities of the drug in the market. The second point was, that the dose should be regulated as carefully as that of any other remedy in disease; and his experience had shown that the acute cases require different doses from the chronic disorders. The swellings of the lip he had merely mentioned incidentally as interesting cases, and ad not based any conclusions upon them. The only original conclusion was that based upon non-parasitic sycosis. The activity of the drug doubtless depends, in a great measure, upon freeing sulphuric acid in the system. He had also used the sulphide of ammonium, but it is more variable and not permanent, although in some cases decidedly more effective.

Adjourned.

[TO BE CONCLUDED IN DECEMBER NUMBER]

